# A comprehensive investigation of negative visitor behaviour in the zoo setting and captive animals' behavioural response

**DOI:** 10.1016/j.heliyon.2023.e16879

**Published:** 2023-06-03

**Authors:** Courtney Collins, Sean McKeown, Ruth O’Riordan

**Affiliations:** a School of Biological, Earth and Environmental Sciences and the Environmental Research Institute, University College Cork, Ireland; b Fota Wildlife Park, Carrigtwohill, Co. Cork, Ireland

**Keywords:** Zoo, Visitors, Negative behaviour, Animal behaviour, Circuit-study

## Abstract

Negative visitor behaviour in zoos such as banging, shouting and feeding animals are unwanted, but under-studied, visitor actions. It is not known how prevalent negative behaviour is, which species or enclosure type receives the most negative behaviour or how these behaviours affect zoo-housed animals. In this study, a comprehensive assessment of negative visitor behaviour, using an innovative methodology, was conducted at 25 different enclosures at Fota Wildlife Park, Ireland. Additionally, animal activity level and out of sight behaviour was observed. Descriptive statistics and general linear models were used to investigate which variables affected behaviour. Banging was the most common negative behaviour, while Humboldt penguins, lion-tailed macaques and Sumatran tigers were the most harassed species. Negative actions increased as visitor number increased and at traditional-style viewing areas. Active animal behaviour and out of sight animals were effected as negative visitor behaviours increased, but there appeared to be a tolerance threshold before a behavioural response was observed. By understanding negative behaviours, zoos can strive to reduce them and promote positive animal welfare.

## 1 Introduction

As peoples' connection to nature and wildlife declines with growing urbanisation [1], zoos are an increasingly important venue for visitors to engage with nature and see wild animals up-close [2,3]. In fact, many visitors report experiencing positive emotional connections with captive animals during their visit to a zoo [4,5]. Emotional connections can lead to empathy for conspecifics in the wild, which has been described as an essential step in motivating pro-conservation behaviour [[6], [7], [8]]. Positive zoo experiences often originate from viewing active animals or having an up-close encounter with an animal [9,10]. Zoos can facilitate these interactions through controlled guest experiences such as feeding, petting and public animal training [[11], [12], [13]]. Visitors report that they have a stronger sense of connection to an animal, if they perceive that the animal is paying attention to them or even other visitors [5]. Reportedly, a third of zoo visitors have an ‘extra special’ zoo experience, such as making eye contact with an animal or observing animals playing at an exhibit [10]. However, given the importance of connections in establishing empathy and positive behaviour [4], this is not a high figure.

Consequently, if animals are not easily visible or active, visitors may attempt to force connections with unsuitable, uncontrolled interactions. When negative visitor behaviour occurs, such as banging on glass or shouting, this may be the visitors' attempt to elicit a response from the animals [14,15]. Additionally, some visitors may presume that zoo animals are trying to interact with them; however, animal behaviour may be misconstrued by visitors who observe their behaviour through an anthropomorphic lens [16]. For example, an up-close encounter that a visitor considers a positive emotional experience, such as making physical contact or eye contact, could be stressful or harmful to an animal [17]. Forming connections with animals is important for visitors [4]; however, in zoos, the animals' welfare is paramount. With a vast range of species housed in zoos and over 700 million visitors annually [18], the scope for potential animal-visitor encounters is interminable. However, almost no systematic research exists in the area of unregulated, unsolicited visitor behaviour while viewing captive animals.

Previous research has shown that zoo visitors are drawn to charismatic megafauna [19]; and species type may affect empathy [16]. However, there is no indication if visitor behaviour while viewing these animals differs from less popular species. Enclosure type is also known to affect visitors' attitude while viewing animals [20]. Naturalistic and free-ranging exhibits provide more enjoyment, better learning opportunities and engender more respect for the animals than viewing traditionally housed animals [[21], [22], [23]]. While free-ranging ‘exhibits’ provide the ultimate freedom for captive animals, they potentially expose the animals to more intense interactions with the public, who might attempt to chase, touch or even feed free-ranging animals [24]. There is an almost complete lack of research quantifying visitors' behaviour towards animals at different exhibit types.

On the opposing side of this dynamic relationship are the animals. Zoo-housed animals must cope with visitors on a daily basis. The effect of visitors on captive animals has been well-studied and summarized as three alternative hypotheses; visitors may have either a positive (enriching), a neutral, or a negative (potentially deleterious to welfare) influence on animals [25]. Mitchell et al. [26] found that visitors who viewed animals passively may be far less disturbing than visitors who engage in active or negative behaviour. For example, primate species were more likely to direct behaviour at active visitor groups than passive ones and locomotory behaviour was higher when active visitor groups were present [26]. Loud visitor sounds such as banging and shouting could provoke fear or aggression and lead to a reduction in welfare over time [[27], [28], [29], [30]]. Uncontrolled feeding attempts, which may be perceived as a positive interaction by animals and visitors, also have the ability to severely affect captive animals' health and welfare and could endanger visitors [30,31].

Negative visitor behaviour has been described as both common and atypical in the zoo, showing the need for accurate quantifiable research [27,32,33]. Currently, most evidence of negative behaviour is derived from research that attempts to reduce it [[34], [35], [36]]. In the seminal study by Kratochvil and Schwammer [35], the authors attempted to reduce the frequency of visitors knocking on glass in an aquarium from a baseline of two knocks per 100 visitors by using three different types of signage. The authors report success with the signs but did not consider a subsequent impact in fish welfare. Collins et al. [14] used an educational intervention to alter children’s behaviour as they viewed penguins (*Spheniscus humboldti*) and free-ranging ring-tailed lemurs (*Lemur catta*). The authors observed a reduction in negative visitor behaviour but found no impact on animal behaviour. Similar studies with limited number of species or enclosures have taken place with little penguins (*Eudyptula minor*) and meerkats (*Suricatta suricatta*) where signage, staff and physical barriers were used to control negative visitor behaviour [27,36].

However, there has been no comprehensive investigation into how common unregulated, negative behaviour is or which type of enclosure or species receives the most negative behaviour even though this could have a direct impact on captive animal welfare. The current research is an investigation of negative visitor behaviour at 25 animal enclosures at Fota Wildlife Park and each species' behavioural response. The overall objective of the research was to quantify negative visitor behaviour in the zoo. Specifically, the research aimed to determine which animals, in which type of enclosures, received the most negative behaviour and which type of negative behaviour was the most common. Additionally, the study sought to investigate which visitors were most likely to engage in negative behaviour and finally to determine if there was a behavioural response from the animals observed. It was predicted that charismatic megafauna would be the most ‘harassed’ species, because they are generally the most popular with visitors [19]. Furthermore, it was thought that there would only be a limited behavioural response from the animals, since many animals do not react with an overt behavioural response to visitors [25] and only a restricted range of behaviours were observed. It was thought that this would be most likely to occur when large crowds were present, and that children would be most likely to engage in these actions.

## 2 Materials and methods

### 2.1 Study site and animals

The research was carried out at Fota Wildlife Park (Fota), Carrigtwohill, County Cork, Ireland. It included 24 species in 25 different enclosures (Table 1). The study was conducted over 13 days between July and August 2020. Data collection occurred between zoo closures due to the Covid 19 pandemic. The daily visitor figures were down slightly at Fota from pre-Covid times; however, zoo staff communicated to the primary research that they still considered the zoo to be very busy during the research period. The average daily number of visitors at the park during the study was 3230 visitors. Certain enclosures had visitor restrictions in place to avoid overcrowding; therefore, data were not collected in these areas. Observations did not take place during inclement weather therefore the weather was generally dry and mild with an average daily temperature of 18 °C. The study included visitors at Fota who were visiting the wildlife park on the days that the research occurred. This research involved the unobtrusive observation of visitors where no personal or identifying information was collected. This research received ethical approval from Fota Wildlife Park’s Research Ethics Committee.

**Table 1:** Description of the species and visitor observations included in the study.

Species including scientific name	Enclosure type∗	Animals per enclosure	Mean number of visitors per observation session∗∗	Total number of negative Behaviours observed	Mean number of negative Behaviours observed ±SE
All species	NA	6 (mean)	11	146	0.23 ± 0.10
1. Meerkat (*Suricatta suricatta*)	Traditional	4	8	4	0.16 ± 0.07
2. Black and white colobus monkey (*Colobus guereza kikuyuensis*)	Traditional	3	4	4	0.16 ± 0.09
3. Cheetah (*Acinonyx jubatus*)	Naturalistic	4	14	1	0.04 ± 0.04
4. Rothschild’s giraffe (*Giraffa camelopardalis rothschildi*)	Naturalistic	9	13	2	0.08 ± 0.06
5. European bison (*Bison bonasus*)	Naturalistic	14	11	5	0.20 ± 0.08
6. Asiatic lion (*Panthera leo persica*)	Traditional	7	8	9	0.36 ± 0.23
7. Indian rhinoceros (*Rhinoceros unicornis*)	Naturalistic	3	22	2	0.08 ± 0.06
8. Lar gibbon (*Hylobates lar*)	Island	4	17	2	0.08 ± 0.06
9. Sumatran tiger (*Panthera tigris sumatrae*)	Naturalistic	3	19	11	0.44 ± 0.14
10. Lion-tailed macaque (*Macaca silenus*)	Traditional	18	9	15	0.60 ± 0.18
11. Visayan warty pig (*Sus cebifrons*)	Naturalistic	2	6	3	0.12 ± 0.07
12. Red panda (*Ailurus fulgens*)	Naturalistic	4	14	10	0.40 ± 0.14
13. Howler monkey (*Alouatta caraya*)	Island	4	2	2	0.08 ± 0.08
14. Humboldt penguin (*Spheniscus humboldti*)	Naturalistic	26	20	18	0.72 ± 0.22
15. Spider monkey (*Ateles fusciceps rufiventris*)	Island	8	17	4	0.16 ± 0.07
16. Ring-tailed lemur (*Lemur catta*)	Traditional	7	8	6	0.24 ± 0.10
17. White-faced saki monkey (*Pithecia pithecia*)	Traditional	4	9	7	0.28 ± 0.11
18. Siamang gibbon (*Symphalangus syndactylus*)	Island	4	17	2	0.08 ± 0.06
19. Grey-cheeked mangabey (*Lophocebus albingena*)	Island	3	6	1	0.04 ± 0.04
20. Chilean flamingo (*Phoenicopterus chilensis*)	Island	5	5	1	0.04 ± 0.04
21. Drill (*Mandrillus leucophaeus*)	Traditional	5	7	10	0.40 ± 0.18
22. Harbour seal (*Phoca vitulina*)	Traditional	2	11	7	0.28 ± 0.09
23. Cheetah (*Acinonyx jubatus*) mother with three cubs	Naturalistic	4	12	8	0.32 ± 0.10
24. Grant’s zebra (*Equus burchelli boehmi*)	Naturalistic	5	6	2	0.08 ± 0.08
25. Brazilian tapir (*Tapirus terrestris*)	Traditional	7	9	10	0.40 ± 0.10

∗ This refers to the type of viewing area where the observations took place but does not describe the entire enclosure.

∗∗ Rounded to the nearest whole number.

### 2.2 Procedure

**Fig. 1:**
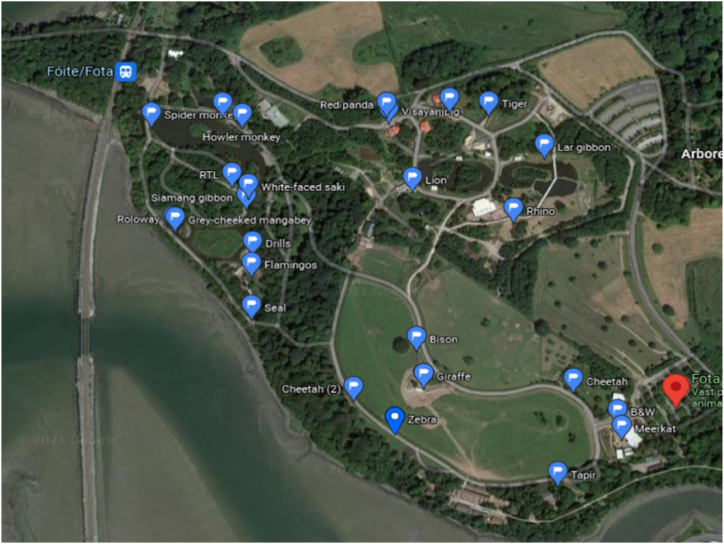
Satellite map of Fota Wildlife Park, where the red marker is the approximate start and finish of the circuit and each blue flag represents a fixed point on the circuit. (Photo source: Google Maps). (For interpretation of the references to colour in this figure legend, the reader is referred to the Web version of this article.)

Animal and visitor behaviour data was collected using an innovative methodology. Similar to the methods described by Harley et al. [37], the researcher, dressed in plain clothes, walked two uni-directional circuits or ‘loops’ of Fota, one in the morning (circuit 1) between approximately 11:00–13:00 and the second (circuit 2) in the afternoon between approximately 14:00–16:00. Generally, two circuits were completed each day, but for logistical reasons on a few days only one circuit was completed. This yielded a total of 25 circuits, which lead to 25 different observations at each species. Each circuit followed the same route and the researcher stopped along the pathway at each of the predesignated animal enclosures at a fixed point (Fig. 1) [37]. The fixed points were determined based on the best vantage point and animal enclosure use, which was discovered during preliminary research and marked using Google maps. Species inclusion was based on feasibility, including access from the route and visibility. The circuit was approximately 5 km long and took approximately 2 h to complete.

At each fixed point on the circuit, the researcher stopped to observe and record visitors' and animals' behaviour. Due to the size and complexity of the enclosures, the researcher moved up to 10 m in any direction from the fixed point to make accurate observations. Generally, the enclosures at Fota Wildlife Park are large and multifaceted with naturalistic features and animals have access to indoor and outdoor spaces. For example, the central mixed-species exhibit is over 11 acres. Therefore, for consistency, observations took place at a fixed viewing point at each enclosure which may have included traditional features. For instance, the drill enclosure includes a large outdoor island, but viewings occurred at a traditionally glass fronted window section of the enclosure and thus the habitat is described as traditional (Table 1).

Using a modified version of instantaneous scan sampling [38], when the researcher arrived at each fixed point on the route the number of animals which were active, inactive and out of sight, as well as the total number of visitors present at the enclosure and the number of negative behaviours that occurred were recorded. Additionally, the age (child or adult), gender (male or female) and social grouping (alone, social or family) of the visitor(s) that engaged in the negative behaviour was recorded. Ideally, individual visitor behaviour would have been tracked; however project logistics and ethical protocols made this impossible. Visitor demographic categories were decided based on the researcher’s best estimation. Negative behaviours were considered as any actions that were not compliant with the rules of Fota Wildlife Park (Table 2). A series of shouts or bangs on the enclosure by the same visitor were only counted once, unless separated by five or more seconds. This procedure took approximately two to 3 min to complete at each enclosure. Because of the variable nature of the zoo and the visitors, it was not possible to standardise the time spent at each enclosure.

**Table 2:** Negative visitor behaviours included in the study at Fota Wildlife Park.

Negative behaviour	Description
Bang on glass or other parts of enclosure	Tap, bang, kick or knock on glass or surrounding structures of enclosure with a hand, foot, stick or other object.
Shout	Using a raised voice, directing a yell, scream or shout at an animal
Throw	Throwing rocks, sticks, paper or other materials into an enclosure or at an animal
Touch	Making physical contact with an animal including, poking, rubbing or picking the animal up
Feed	Offering or giving a food item to an animal or throwing food into an enclosure.
Enter enclosure	Enter all or part of an enclosure with an arm leg, etc. or an object such as an umbrella or a stick, crossing a barrier with more than 50% of body.

In addition to the instantaneous scan samples that occurred at fixed points along the circuit, the researcher also used ad libitum sampling to record any negative visitor behaviours that were observed anywhere along the path, including any incidence of negative behaviour with a free-ranging species [38]. The free-ranging species at Fota are mostly avian, including waterfowl (e.g. Anatidae) and Indian peafowl (*Pavo cristatus*), but also, Bennett’s wallabies (*Macropus rufogriseus*) and Eastern grey kangaroos (*Macropus giganteus*). The number of negative behaviours observed along the route and with free-ranging species were counted per circuit throughout the study.

### 2.3 Data analysis

The primary objectives of the analysis were to test 1) which variables effected negative visitor behaviour and 2) which variables effected two animal behaviours (Table 3). First, data are presented using descriptive statistics (Table 1, Table 4). Preliminary investigation indicated that circuit number (time of day) did not affect visitor behaviour and that daily visitor number and the number of visitors present at exhibit were highly correlated. Therefore, daily visitor number and circuit number were eliminated from further analysis.

**Table 3:** Variables included in the three statistical models.

Visitor behaviour	
Dependent variable	Independent variables
1). Total frequency of negative visitor behaviour	Species type (categorical variable)
	Enclosure type (categorical variable)
	Number of visitor present (continuous variable)
**Animal behaviour**	
**Dependent variable**	**Independent variables**
2). Proportion of active animals	Species (categorical variable)
3). Proportion of animals out of sight	Number of visitors present (continuous variable)
	Frequency of negative visitor behaviour (continuous variable)

**Table 4:** Negative visitor behaviours and the demographics of visitors who engaged in the behaviours represented as the proportion of observations (rounded to the nearest decimal place) to occur at Fota Wildlife Park.

Visitor behaviour	Proportion∗
Bang	0.53
Shout	0.12
Throw	0.04
Touch	0.02
Feed	0.03
Cross barrier/enter enclosure	0.24
Other	0.02
**Age**	**Proportion**∗∗
Adult	0.10
Child	0.80
Mix	0.10
**Gender**	**Proportion**∗∗
Male	0.63
Female	0.26
Mixed	0.11
**Group type**	**Proportion**∗∗
Alone	0.02
Family	0.94
Social (Adults)	0.03
Mixed	0.02

*** Note, No data is available regarding the overall proportions of visitors in each demographic group. While it is possible that the above findings reflect the general composition of the sample, it is considered unlikely that the ratios of children to adults, and males to females, would be so high.

∗ Out of 146 individual incidence of negative Behaviours.

∗∗ Out of 115 observed negative occurences.

Negative visitor behaviour is presented as the total occurrence of negative behaviours observed. However, it was not possible to observe a full range of animal behaviours for each species in this study. Therefore observations were limited to the proportion of animals during each scan sample that were active, inactive or out of sight. For the purpose of data analysis, because inactive and active animal behaviours are inversely related, it was decided to focus on active behaviour and out of sight. Previous research has shown that visitors may be drawn to active animals [39,40], but equally important is the concept that animals may avoid visitors or hide in aversive situations [28,41]. This scenario can lead to a confound between these two alternative hypotheses that has proven difficult for researchers to disentangle – visitors are drawn to active animals who may in turn hide from large or noisy crowds of visitors [25,42]. However, these two behavioural responses by animals are still appropriate indicators of response to visitors in this study. Where possible, inferences have been made to disentangle the directionality of these behaviours with acknowledgement that this was not specifically tested for in this study.

Next, data were assessed for normality using plotted histograms and the Kolmogorov-Smirnov test. Visually, data appeared near normal. Therefore, to test which independent variables affected visitor behaviour and animal behaviour, general linear models were constructed to model occurrence of negative visitor behaviour and two animal behaviours against several independent variables (Table 3). Validation of the models was tested by plotting a histogram of residuals, plotting the residuals against the fitted values and checking linearity of the model. Independent variables were tested for multicollinearity and were found to be below the variance inflation factor (VIF) tolerance level of 2.5 in all cases. However, because of an imbalance in the independent variables, the variance was not homogenous across the residuals. To correct for heteroscedasticity, a weighted least squares (WLS) multiple regression was used in all cases. A preliminary investigation of these data, using the original model, did not report any statistically significant interactions, or, because of the aforementioned data imbalance, it was not possible to compute interactions. Therefore, the final models did not include interaction terms. Data analysis was conducted using SPSS version 26. The accepted alpha level for these analyses was p < 0.05.

## 3 Results

### 3.1 Descriptive statistics

In total, 625 individual observations took place over 13 days at 25 different enclosures. Daily visitor number ranged from 2188 to 3973 ($\bar{x}$ = 3424 ± 19.08SE, information provided by Fota Wildlife Park), while the number of visitors present at each enclosure ranged from 0 to 55 ($\bar{x}$ = 11 ± 0.32SE) (Table 1). The most visited species included the rhinoceros, the Humboldt penguins and the Sumatran tigers, while the least visited were the howler monkeys, the black and white colobus and the mangabeys (Table 1).

**Fig. 2:**
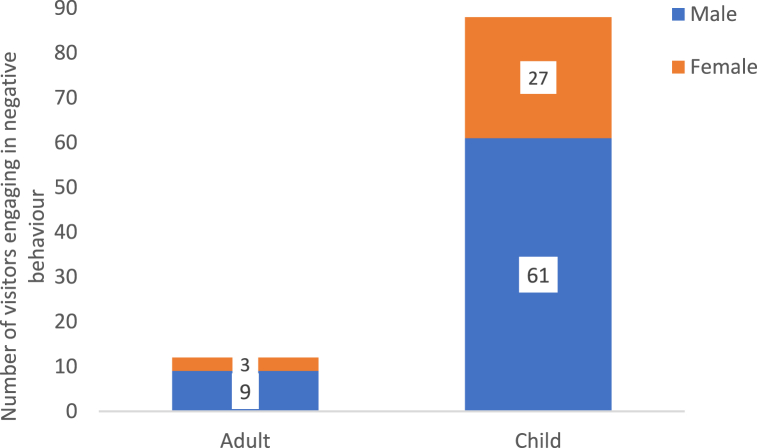
The total number of negative behaviours represented as categories Adult vs Child, further divided by gender. Note: n = 100 because it was not possible to identify the demographics of all visitors engaging in negative behaviours.

Out of 625 observations at fixed points on the circuit, a negative behaviour(s) occurred during 115 observations (18.4%); at times this included more than one negative behaviour. This yielded a total of 146 negative visitors behaviours (Table 1). A negative behaviour was observed at least once at every enclosure included in the study. The species to receive the most incidences of negative behaviour were the Humboldt penguins, the lion-tailed macaques and the Sumatran tigers, whereas the fewest negative incidence occurred at the cheetahs (without cubs), the mangabeys and the flamingos (Table 1). Banging was the most prevalent negative behaviour followed by crossing a barrier or entering an enclosure (Table 4). Children, male visitors and family groups were most likely to engage in negative behaviour, although family groups likely comprised the majority of visitors to Fota (Table 4). Of the children who engaged in negative behaviour 69% were male and for adults visitors who engaged in negative behaviour 75% were male (Fig. 2).

Ad libitum sampling revealed 58 total incidences of negative behaviour beyond the regular sampling points and 25 occurrences of negative behaviour were observed with free-ranging animals, observationally this was mostly avian species.

### 3.2 Visitor behaviour

**Fig. 3:**
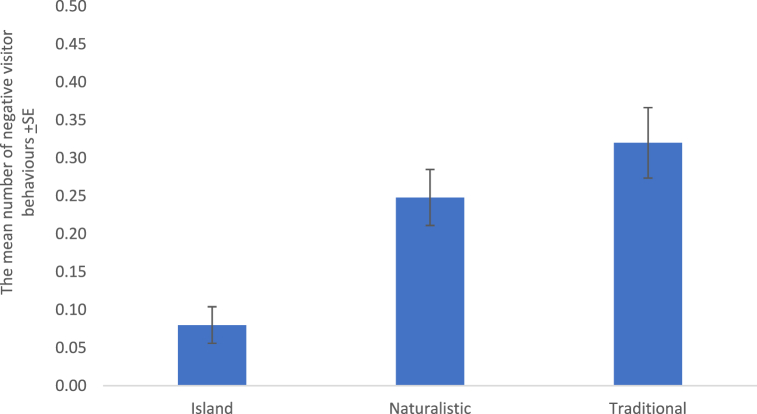
Negative visitor behaviours observed at three different types of animal exhibits at Fota Wildlife Park.

The results of the weighted least squares regression model (Table 3) (F(3,621) = 28.967, p < 0.001, r^2^ 0.123) revealed that species type (p = 0.013, β = 0.105), the number of visitors present at the enclosure (p < 0.001, β = 0.289) and enclosure type (p < 0.001, β = 0.175) affected negative visitor behaviour. Negative behaviour was more likely to occur at certain species, including Humboldt penguins, lion-tailed macaques and Sumatran tigers (Table 1), as visitor number increased and at traditional enclosures (Fig. 3).

### 3.3 Animal behaviour

**Fig. 4:**
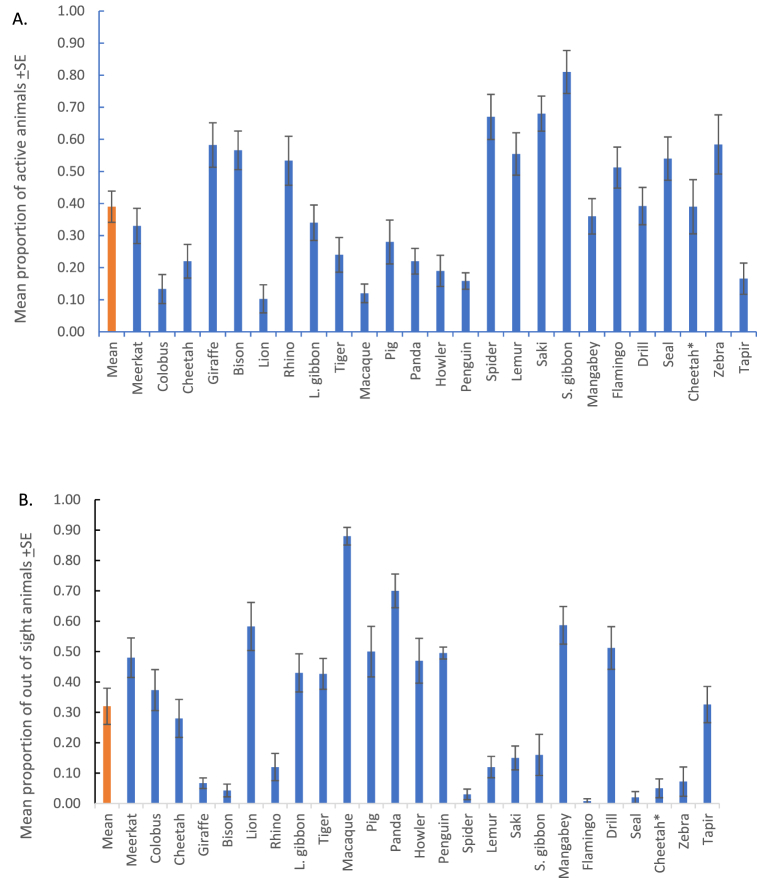
Mean proportion of the number of A) active animals and B) out of sight animals for each species observed at Fota Wildlife Park. The first column in each figure represents the combined total of the proportion of active animals or out of sight animals. Species names have been abbreviated (Table 1 for full scientific names).

**Fig. 5:**
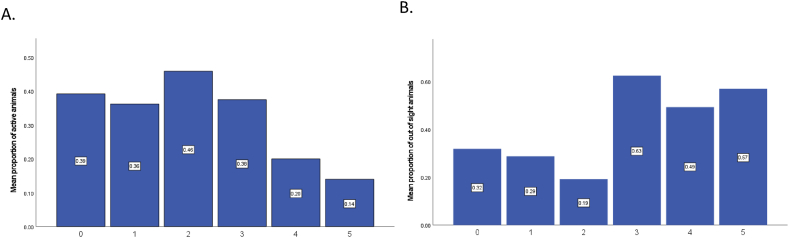
The mean proportion of A) active animal behaviours and B) out of sight animals during the different frequencies of observed negative visitor behaviours.

The results of the weighted least squares regression model (Table 3) for active animal behaviour (F(3,621) = 25.688, p < 0.001, r^2^ 0.110) revealed that species type (p < 0.001, β = 0.194), the number of visitors present (p < 0.001, β = 0.290), and the frequency of negative visitor behaviour (p = 0.027, β = −0.086) affected animal activity. Animals most likely to display active behaviour were all primates including the Siamang gibbons, the white-face saki and the spider monkeys (Fig. 4A). Additionally, active animal behaviour was associated with an increase in visitor numbers. The effect of negative visitor behaviour on active animal behaviour is more difficult to interpret. The negative Beta value indicates that active animal behaviour decreased as negative visitor behaviours increased. However, this trend is not observed until four or more negative visitor behaviours occurred (Fig. 5A).

The model for the proportion of animals out of sight (Table 3) (F (3,621) = 362.415, p < 0.001, r^2^ 0.636) showed that species type (p < 0.001, β = 1.695), the number of visitors present (p < 0.001, β = 1.444), and the frequency of negative visitor behaviour (p < 0.001, β = −0.693) were significant predictors of animal behaviour. Animals with the highest mean number of individuals out of sight were the lion-tailed macaques, the red pandas and the grey-cheeked mangabeys (Fig. 4B). Although a positive Beta value is reported in the model for the number of visitors present, a visual inspection of out of sight animals plotted against visitor number showed that as the number of visitors increased, the proportion of out of sight animals decreased. The Spearman rank test also revealed a negative correlation between visitor number and out of sight animals (rs = −0.245; p < 0.001). This is discrepancy is likely due to the effect of multiple variables in the model and other untested variables, which are not controlled for in the simple correlation [43]. In this case, the sign of the regression coefficient (β) is the correct interpretation, indicating that out of sight animals increased as visitor number increased [44]. The proportion of out of sight animals was also affected by the frequency of negative visitor behaviour, but only when more than three negative visitor behaviours occurred (Fig. 5B).

## 4 Discussion

Despite serious implications, negative visitor behaviour in the zoo is a highly under-investigated area. In fact, this is the first study to quantify negative visitor behaviour at a large number of animal exhibits, using a novel and robust methodology. The findings from this study at a wildlife park revealed that negative behaviour is not uncommon and occurred in approximately 18% of observations.

The most common negative behaviour to occur was banging. This concurs with a prior study that found that approximately 22% of visitors at a great ape house knocked on the glass, but overall spent limited time engaged in this activity [33]. Since several previous studies have implemented changes to reduce visitor banging [14,35,36], it can be inferred that banging is a perceived problem in zoos and aquariums. The results of this study empirically confirm that. However, the impact of banging on captive animals and the precise reason (noise, activity, proximity) that banging may be disturbing should be teased out in future research.

The potentially serious consequences of visitors crossing barriers in zoos, and the fact that 24% of negative actions in the current study involved people crossing barriers could have important implications. For example, the now famous story of, Harambe, a western lowland gorilla who was shot dead by zoo staff, when a child crossed a barrier and fell into his enclosure, shows the most severe implications of visitors breaching barriers [45]. Although Fota Wildlife Park does have many staff present throughout the zoo, staff cannot observe and correct every time that a visitor crosses a barrier. Therefore, awareness of the prevalence of these incidences and enclosure design that minimises the possibility of visitors entering enclosures is essential for visitor and animal safety.

Previously it was speculated that children were responsible for much of the negative behaviour that occurs in zoos and aquariums [35], however this is the first study to offer evidence of this. Since children make up a large part of the zoo audience [46], this result has important implications regarding how zoos engage with their younger visitors. It may be that more child appropriate messaging, including signage and education [14] is requried to stop these unwanted behaviours. Although the overall number of adults engaging in negative behaviour is low (12 out of 115 observed occurrences), results found here indicate that men rather than women are more likely to engage in negative behaviour (9 of the 12 adult occurrences were attributed to men). This supports previous research by Myers et al. [5] that found that women are more likely to respond to zoo animals with empathy and emotion than men. Girls are also reported to learn more during a zoo visit than boys [47,48]. The difference between the genders in response to zoo animals is useful information for modifying visitor behaviour and suggests that zoos may need to target their messaging at specific groups and appeal to their male audience, but more research with larger samples is needed.

Family groups (94%) were by far the most common type of visitor group to engage in negative behaviour. Although it was not possible to specifically measure the total proportion of visitors in family groups, it is likely that families are the most common type of visitor at Fota so this is not an unexpected result. Clayton et al. [49] state that sharing emotional experiences at the zoo provides validation for the visit and produces a common memory, which builds social relationships. It could be that family groups are creating memories and common emotional experiences by attempting to engage with the animals. Equally family groups could be responding to social norms and societal pressure to engage with animals [50]. Previously an educational intervention for children was used to successfully mitigate negative viewing behaviour [14]. This type of hands-on educational activity could also be extended to family groups, where normative beliefs towards animals could be discussed together.

While prior studies have found that certain species are subject to negative visitor behaviour such as the great apes [33,42], meerkats [36] and penguins [27], there has been no comprehensive overview of which species in zoos are likely to receive the most negative visitor behaviour. The results from this study show that the Humboldt penguins, the lion-tailed macaques and the Sumatran tigers received the highest number of negative incidences. The tigers and penguins also attracted the highest number of visitors. Since the current research discovered that negative behaviour was more likely to occur with higher numbers of visitors, this finding is unsurprising. Felid species, penguin species, and primates are also amongst the most charismatic and ‘liked’ zoo species [19,47,51]. The fact that the current research found that the most ‘harassed’ species are also the most visited species, could be evidence that visitors are simply looking for connections with their favourite animal and are not intending to cause harm. Regardless, the animals' welfare is paramount; however, when appropriate zoos could facilitate opportunities for connections with known favourites such as the visitor-tiger environmental enrichment activity implemented by Collins et al. [52]. Furthermore, zoos should be aware of the likelihood of negative events at popular exhibits as crowd size increases.

The least harassed species were also generally unpopular, particularly the mangabeys. The flamingos also received few visitors and few negative behaviours, which supports previous research that birds (except penguins) are unpopular with zoo visitors [53]. Interestingly, the cheetah without cubs received similar visitor numbers but far fewer negative behaviours than the cheetah with cubs, confirming visitors' attraction to young animals [53] which may result in unregulated interaction attempts. Also, the cheetah without cubs are situated next to the entrance to the zoo. The impact of exhibit location on visitor number and behaviour was not evaluated in this study, but may affect visitor actions and should be considered in future research.

Exhibit design influences visitors' perceptions of animals. Davey [54], Moss et al. [22] and Tofield et al. [55] all confirm that visitors spent longer at and prefer to see animals in enriched third-generation exhibits. Broadly, naturalistic enclosures command more respect and better attitudes from the public than sterile cage-like exhibits [20,21,56,57]. This supports the findings from the current study that negative behaviour was observed more frequently at enclosures with traditional viewing areas. Negative actions, specifically banging, could be opportunistic. At traditional enclosures, with glass viewing windows, it is simply easier for visitors to make noise by banging on the glass or a metal frame. However, Sommer [58] suggests that traditional exhibits can lead to disrespect and indifference towards animals. It is possible that non-naturalistic displays send negative messages to visitors about the animals housed there [59], while naturalistic displays encourage visitors towards empathy and respect [9]. In this study, some of the exhibits were categorized as traditional because of the arrangement of the viewing window. However, most of the displays in their entirety were naturalistic, indicating that negative behaviour is indeed opportunistic rather than brought on by disrespect towards animals because of the environment of their enclosure.

It is perhaps surprising that only 25 incidences of negative behaviour, mostly involving birds, were observed with the free-ranging animals at Fota. This supports previous research at Fota with the free-ranging ring-tailed lemurs that found visitors attempted to interact with lemurs in less than 1.4% of observations [60]. Interactions between visitors and free-ranging animals are uncommon, though Fota does have staff present to monitor its free-ranging animals, which may dissuade negative behaviour. Zoos must be aware of the implications of different enclosure types on visitors' behaviour and strive to provide exhibits that support best-practice for the animals, but also send positive messages to visitors.

Captive animals are known to react to the presence of zoo visitors [25,30]. Increasing visitor numbers are associated with stress for some species (e.g. Western lowland gorillas [61]), increased noise levels [29], and the current study confirms that higher visitor numbers are also related to more incidence of negative visitor behaviour. Zoos must achieve a delicate balance between animal welfare, visitor enjoyment and financial concerns so that limiting visitor numbers is not a viable option for zoos [62]. However, zoos should be aware that with increasing visitor numbers comes increasing noise and negative behaviour. At the busiest times, zoo could ensure that staff are present to control crowds.

It is well known from previous research that visitors like to see easily visible animals engaging in active behaviour [9,20,62]. This research confirms that higher visitor numbers were also associated with increased animal activity. It is probable that larger crowds of visitors were attracted to the active animals; however, it is also possible that the animals were stimulated into activity when more visitors were present. This conundrum highlights the visitor attraction theory described by Margulis et al. [40]. Although directionality was not specifically tested in this study, the positive correlation between visitor number and animal activity likely supports the visitor attraction theory. Statistical anomalies make interpreting the correlation between visitor number and out of sight animals challenging, but when other significant variables such as species type are controlled for, it is likely that the number of out of sight animals increased as visitor number increased. Further research is needed to clarify this, but data indicates that the animals in this study retreated (out of sight) with increasing numbers of visitors, which has been reported in other studies [24,27]. Though not approved for the current research, video may help to tease out directionality.

The statistical findings from this research also indicate that the proportion of active animal behaviour and out of sight animals both decreased as negative behaviour increased. However, confounds in the data occurred, and it is likely these results are also impacted by individual species differences [25,30]. Visual inspection of these data by the number of negative visitor actions, suggests that there is a tolerance threshold before a behavioural response to visitors occurred, which has been reported in previous studies with captive birds and gorillas [63,64]. The proportion of active animals only appeared to diminish when four or more negative behaviours occurred and after decreasing with two negative actions out of sight increased after three or more negative behaviours occurred. This means that more incidence of negative behaviour were the most disturbing to these animals.

The association between negative visitor behaviour and higher visitor numbers is also an important consideration. For example, when animals are active visitor numbers increase, but higher visitor numbers are also associated with an increase in negative visitor behaviour. Additionally, it is likely that when the animals were out of sight some visitors may have attempted to attract their attention by banging on the glass. In fact, the lion-tailed macaques were one of the least visible, yet most harassed species in the study, that are also housed in a traditionally glass fronted enclosure, albeit with plenty of space and complexity. Previous research on this macaque group, which occurred after they moved into this new, larger, more complex enclosure, in closer proximity to visitors, did find that the primates spent more time out of sight than in their previous enclosure, which the authors attribute to dense vegetation and increased exploratory behaviour [65]. Conversely, the mangabeys were often out of sight, but were also not popular and housed on an island, and received amongst the least negative behaviour of all species. However, it should not be overlooked that as negative visitor behaviour increased the animals may have attempted to hide. Yet, there is no substantial behavioural evidence that the animals' welfare was compromised by visitor behaviour in this study. The limited animal behaviours included here should be expanded in future studies to include a range of behaviours which would allow for a more comprehensive evaluation of the animals' welfare.

Further in-depth investigation in this area is required to disentangle the behavioural and statistical complexities discovered here. The findings of this research suggest that it is likely the visitor viewing area, together with crowd size, species and animal activity level that influences visitor behaviour. Future research in this area should focus on these variables as known contributors to negative visitor behaviour. The statistical tests used here did not allow for interactions to be considered, though they may be important in these findings and likely lead to some of the anomalies uncovered here. Furthermore, the results of the models show that several statistically significant relationships occurred such as between the number of visitors and negative visitor behaviour. However, the low r^2^ value suggests that there are other significant predictors of behaviour that were not tested for. It is likely that personal visitor demographics like personality, education and previous experience with animals contribute to visitor behaviour. It was not possible to test these variables in the current study, but future research should consider other motivators of visitor behaviour.

While every effort was made to standardise the variables included in this study, limited resources, ethical controls and researcher availability, made standardisation and recording of certain variables impractical. For example, because of the availability of only one researcher and large crowds of visitors, it was not possible to count the exact number of visitors present during each negative visitor behaviour. Nor was it possible to standardise the time spent at each exhibit, given the variability of each enclosure and fluidity of visitor movements. Future research, should build on this novel study by conducting preliminary research to standardise the time requried to collect all of the required data within a specific time frame e.g., 3-min. Additionally, the presence of a second researcher would aid with logistics, such as timing with a stopwatch and observing either animal or visitor behaviour. By not standardising these variables, it is possible that certain results are inflated if the researcher routinely spent 2 min at one species enclosure and 3 min at another. To the best of our knowledge, this did not occur, but standardising the time at each enclosure would improve future results.

Furthermore, if possible, the total number of men, women, children and families visiting the zoo should be recorded in future studies so that the proportion of visitors who engage in negative behaviour can be adequately analyzed. Recent research has shown that instantaneous sampling is effective for measuring event frequency [66]. However, given the large-scale, multi-species data collection procedure used in the current study, where continuous recording was not possible, inevitably certain negative events will be missed. Over time this may lead to a significant number of negative events not being recorded. Following ethical guidelines, future studies should consider video recordings to obtain a record of all negative behaviours occurring at each exhibit. Finally, it is important to note that this research was conducted between zoo closures during the Covid 19 pandemic. While there did not appear to be a significant impact on visitor attendance or visitor demographics, this information was not specifically recorded and data may have been affected.

The social atmosphere of zoos combined with close contact with animals and emotional stimulation can lead to social cohesion, but also connectedness to animals and possibly empathy and the development of pro-environmental behaviour [49]. However, the search for a connection may rapidly evolve into visitors attempting to interact with an animal through unwanted, unregulated, negative behaviours. It is essential that zoos are aware of the importance of connections for visitors, yet it may be difficult for zoos to balance visitors' affective responses with animals' wellbeing. Thus, making zoos aware of the needs of their visitors and incorporating them into their messaging, husbandry routines, enclosure design and staff scheduling may facilitate the requirements of both the visitors and the animals. Zoos must be aware of the demographics of all of their visitors and the various social groups that visit zoos and consider if their messaging is universally appealing or if it should be tailored to appeal to specific groups. Additionally, staff-supervised, regulated, interactive activities with animals that visitors want to engage with may decrease unsolicited visitor behaviour. For example, designing enclosures, and especially their viewing areas, in a naturalistic respectful way, and scheduling staff to be present during the busiest times could reduce negative behaviour. The results found here give a glimpse of visitor behaviour across a range of species in an Irish zoo. However, to gain a broader, more generalisable picture of visitor behaviour in zoos, this research should be replicated at different types of zoos and aquariums worldwide. Ultimately, it is the responsibility of zoos to ensure their animals' welfare; understanding the behaviour of the visitors who view the animals is an essential step in this process.

## 5 Conclusions

While not widespread, negative behaviour by visitors occurred often enough in this study to warrant further investigation. Negative behaviour was most often instigated by male children and family groups. It was most likely to occur at traditional glass fronted viewing windows during busy times with charismatic species. Visitors are a vital part of the zoo and have the power to advance the conservation movement. It is important that zoos do not alienate their visitors. However, to promote animal welfare, it is recommended that zoos become more aware of their visitors' behaviour and take steps to mitigate unwanted visitor actions.

## Author contribution statement

Courtney K Collins: Conceived and designed the experiments; Performed the experiments; Analyzed and interpreted the data; Contributed reagents, materials, analysis tools or data; Wrote the paper.

Sean McKeown: Contributed reagents, materials, analysis tools or data.

Ruth O’Riordan: Conceived and designed the experiments; Wrote the paper.

## Data availability statement

Data will be made available on request.

## Additional information

No additional information is available for this paper.

## Declaration of competing interest

The authors declare that they have no known competing financial interests or personal relationships that could have appeared to influence the work reported in this paper

## References

[1] Miller J.R. (2005). Biodiversity conservation and the extinction of experience. Trends Ecol. Evol..

[2] Bruni C.M., Fraser J., Schultz P.W. (2008). The value of zoo experiences for connecting people with nature. Visit. Stud..

[3] Consorte-McCrea A., Fernandez A., Bainbridge A., Moss A., Prévot A.C., Clayton S., Glikman J., Johansson M., López-Bao J., Bath A., Frank B., Marchini S. (2019). Large carnivores and zoos as catalysts for engaging the public in the protection of biodiversity. Nat. Conserv..

[4] Clayton S., Fraser J., Saunders C.D. (2009). Zoo experiences: conversations, connections, and concern for animals. Zoo Biol..

[5] Myers O.E., Saunders C.D., Birjulin A.A. (2004). Emotional dimensions of watching zoo animals: an experience sampling study building on insights from psychology. Curator.

[6] Bexell S.M., Jarrett O.S., Ping X. (2013). The effects of a summer camp program in China on children's knowledge, attitudes, and behaviors toward animals: a model for conservation education. Visit. Stud..

[7] Myers O.E., Saunders C.D. (2002). Animals as links toward developing caring relationships with the natural world. Child. Nat.: Psychol., Sociocult., Evol. Investig..

[8] Skibins J.C., Powell R.B. (2013). Conservation caring: measuring the influence of zoo visitors' connection to wildlife on pro‐conservation behaviors. Zoo Biol..

[9] Godinez A.M., Fernandez E.J. (2019). What is the zoo experience? How zoos impact a visitor’s behaviors, perceptions, and conservation efforts. Front. Psychol..

[10] Luebke J.F. (2018). Zoo exhibit experiences and visitors' affective reactions: a preliminary study. Curator.

[11] Anderson U.S., Kelling A.S., Pressley-Keough R., Bloomsmith M.A., Maple T.L. (2003). Enhancing the zoo visitor’s experience by public animal training and oral interpretation at an otter exhibit. Environ. Behav..

[12] Fernandez E.J., Upchurch B., Hawkes N.C. (2021). Public feeding interactions as enrichment for three zoo-housed elephants. Animals.

[13] Jones H., McGregor P.K., Farmer H.L.A., Baker K.R. (2016). The influence of visitor interaction on the behavior of captive crowned lemurs (*Eulemur coronatus*) and implications for welfare. Zoo Biol..

[14] Collins C., Quirke T., McKeown S., Flannery K., Kennedy D., O’Riordan R. (2019). Zoological education: can it change behaviour?. Appl. Anim. Behav. Sci..

[15] Luebke J.F., Watters J.V., Packer J., Miller L.J., Powell D.M. (2016). Zoo visitors' affective responses to observing animal behaviors. Visit. Stud..

[16] Young A., Khalil K.A., Wharton J. (2018). Empathy for animals: a review of the existing literature. Curator.

[17] Collins C.K. (2018).

[18] Gusset M., Dick G. (2011). The global reach of zoos and aquariums in visitor numbers and conservation expenditures. Zoo Biol..

[19] Albert C., Luque G.M., Courchamp F. (2018). The twenty most charismatic species. PLoS One.

[20] Bitgood S., Paterson D., Benefield A. (1988). Exhibit design and visitor behaviour: empirical relationships. Environ. Behav..

[21] Coe J.C. (1985). Design and perception: making the zoo experience real. Zoo Biol..

[22] Moss A., Esson M., Francis D. (2010). Evaluation of a third-generation zoo exhibit in relation to visitor behavior and interpretation use. J. Interpretation Res..

[23] Price E.C., Ashmore L.A., McGivern A.M. (1994). Reactions of zoo visitors to free‐ranging monkeys. Zoo Biol..

[24] Mun J.S.C., Kabilan B., Alagappasamy S., Guha B. (2013). Benefits of naturalistic free-ranging primate displays and implications for increased human–primate interactions. Anthrozoös.

[25] Hosey G.R. (2000). Zoo animals and their human audiences: what is the visitor effect?. Anim. Welf..

[26] Mitchell G., Tromborg C.T., Kaufman J., Bargabus S., Simoni R., Geissler V. (1992). More on the ‘influence’ of zoo visitors on the behaviour of captive primates. Appl. Anim. Behav. Sci..

[27] Chiew S.J., Butler K.L., Sherwen S.L., Coleman G.J., Fanson K.V., Hemsworth P.H. (2019). Effects of regulating visitor viewing proximity and the intensity of visitor behaviour on little penguin (*Eudyptula minor*) behaviour and welfare. Animals.

[28] Morgan K.N., Tromborg C.T. (2007). Sources of stress in captivity. Appl. Anim. Behav. Sci..

[29] Quadros S., Goulart V.D., Passos L., Vecci M.A., Young R.J. (2014). Zoo visitor effect on mammal behaviour: does noise matter?. Appl. Anim. Behav. Sci..

[30] Sherwen S.L., Hemsworth P.H. (2019). The visitor effect on zoo animals: implications and opportunities for zoo animal welfare. Animals.

[31] Kreger M.D., Mench J.A. (1995). Visitor–animal interactions at the zoo. Anthrozoös.

[32] Davey G. (2006). Visitor behavior in zoos: a review. Anthrozoös.

[33] Lukas K.E., Ross S.R. (2005). Zoo visitor knowledge and attitudes toward gorillas and chimpanzees. J. Environ. Educ..

[34] Dancer A.M., Burn C.C. (2019). Visitor effects on zoo-housed Sulawesi crested macaque (*Macaca nigra*) behaviour: can signs with ‘watching eyes’ requesting quietness help?. Appl. Anim. Behav. Sci..

[35] Kratochvil H., Schwammer H. (1997). Reducing acoustic disturbances by aquarium visitors. Zoo Biol..

[36] Sherwen S.L., Magrath M.J., Butler K.L., Phillips C.J., Hemsworth P.H. (2014). A multi-enclosure study investigating the behavioural response of meerkats to zoo visitors. Appl. Anim. Behav. Sci..

[37] Harley J.J., Rowden L.J., Clifforde L.M., Power A., Stanley C.R. (2022). Preliminary investigation of the effects of a concert on the behavior of zoo animals. Zoo Biol..

[38] Martin P., Bateson P. (2007).

[39] Collins C.K., Quirke T., Overy L., Flannery K., O’Riordan R. (2016). The effect of the zoo setting on the behavioural diversity of captive gentoo penguins and the implications for their educational potential. J. Zoo Aquarium Res..

[40] Margulis S.W., Hoyos C., Anderson M. (2003). Effect of felid activity on zoo visitor interest. Zoo Biol..

[41] Birke L. (2002). Effects of browse, human visitors, and noise on the behaviour of captive orang-utans. Anim. Welf..

[42] Choo Y., Todd P.A., Li D. (2011). Visitor effects on zoo orangutans in two novel, naturalistic enclosures. Appl. Anim. Behav. Sci..

[43] Siegel A.F., Wagner M. (2021).

[44] Falk R., Miller N. (1992).

[45] Mkono M., Holder A. (2019). The future of animals in tourism recreation: social media as spaces of collective moral reflexivity. Tourism Manag. Perspect..

[46] Jensen E. (2014). Evaluating children's conservation biology learning at the zoo. Conserv. Biol..

[47] Collins C., Corkery I., McKeown S., McSweeney L., Flannery K., Kennedy D., O’Riordan R. (2020). An educational intervention maximizes children’s learning during a zoo or aquarium visit. J. Environ. Educ..

[48] Randler C., Baumgärtner S., Eisele H., Kienzle W. (2007). Learning at workstations in the zoo: a controlled evaluation of cognitive and affective outcomes. Visit. Stud..

[49] Clayton S., Luebke J., Saunders C., Matiasek J., Grajal A. (2014). Connecting to nature at the zoo: implications for responding to climate change. Environ. Educ. Res..

[50] Ajzen I. (1991). The theory of planned behavior. Organ. Behav. Hum. Decis. Process..

[51] Skibins J.C., Powell R.B., Hallo J.C. (2013). Charisma and conservation: charismatic megafauna’s influence on safari and zoo tourists' pro-conservation behaviors. Biodivers. Conserv..

[52] Collins C.K., McKeown S., O’Riordan R. (2021). Does an animal–visitor interactive experience drive conservation action?. J. Zool. Bot. Gard..

[53] Carr N. (2016). An analysis of zoo visitors' favourite and least favourite animals. Tourism Manag. Perspect..

[54] Davey G. (2006). Relationships between exhibit naturalism, animal visibility and visitor interest in a Chinese Zoo. Appl. Anim. Behav. Sci..

[55] Tofield S., Coll R.K., Vyle B., Bolstad R. (2003). Zoos as a source of free choice learning. Res. Sci. Technol. Educ..

[56] Reade L.S., Waran N.K. (1996). The modern zoo: how do people perceive zoo animals?. Appl. Anim. Behav. Sci..

[57] Shettel-Neuber J. (1988). Second and third-generation zoo exhibits A comparison of visitor, staff, and animal responses. Environ. Behav..

[58] Sommer R. (1972). What do we learn at the zoo?. Nat. Hist..

[59] Blaney E.C., Wells D.L. (2004). The influence of a camouflage net barrier on the behaviour, welfare and public perceptions of zoo-housed gorillas. Anim. Welf..

[60] Collins C., Corkery I., Haigh A., McKeown S., Quirke T., O'Riordan R. (2017). The effects of environmental and visitor variables on the behavior of free‐ranging ring‐tailed lemurs (*Lemur catta*) in captivity. Zoo Biol..

[61] Carder G., Semple S. (2008). Visitor effects on anxiety in two captive groups of western lowland gorillas. Appl. Anim. Behav. Sci..

[62] Fernandez E.J., Tamborski M.A., Pickens S.R., Timberlake W. (2009). Animal-visitor interaction in the modern zoo: conflicts and interventions. Appl. Anim. Behav. Sci..

[63] Nimon A.J., Dalziel F.R. (1992). Cross-species interaction and communication: a study method applied to captive siamang (Hylobates syndactylus) and long-billed corella (Cacatua tenuirostris) contacts with humans. Appl. Anim. Behav. Sci..

[64] Collins C.K., Marples N.M. (2016). The effects of zoo visitors on a group of Western lowland gorillas Gorilla gorilla gorilla before and after the birth of an infant at Dublin Zoo. Int. Zoo Yearbk..

[65] Newman R., McKeown S., Quirke T., O'Riordan R.M. (2021). The effect of a new enclosure on the behaviour of a large captive group of lion-tailed macaques *Macaca silenus*. J. Zoo Aquarium Res..

[66] Brereton J.E., Tuke J., Fernandez E.J. (2022). A simulated comparison of behavioural observation sampling methods. Sci. Rep..

